# Three‐dimensional representation of microdontia of the maxillary third molar

**DOI:** 10.1002/ccr3.867

**Published:** 2017-03-06

**Authors:** Toshiko Inoue, Makoto Saito, Fumio Nishimura, Takashi Miyazaki

**Affiliations:** ^1^ Division of Biomaterials and EngineeringDepartment of Conservative DentistryShowa University School of Dentistry1‐5‐8 Hatanodai, Shinagawa‐kuTokyo142‐8555Japan

**Keywords:** Anomalies, computed tomography, microdontia, teeth

## Abstract

Dentists and maxillofacial surgeons may occasionally encounter various dental anomalies in number, shape, size, eruption, etc. In particular, microdontia is relatively rare. Computed tomography during clinical dental examination is essential for early detection of these anomalies.

Question: What is this condition in Figure 1A ?

**Figure 1:**
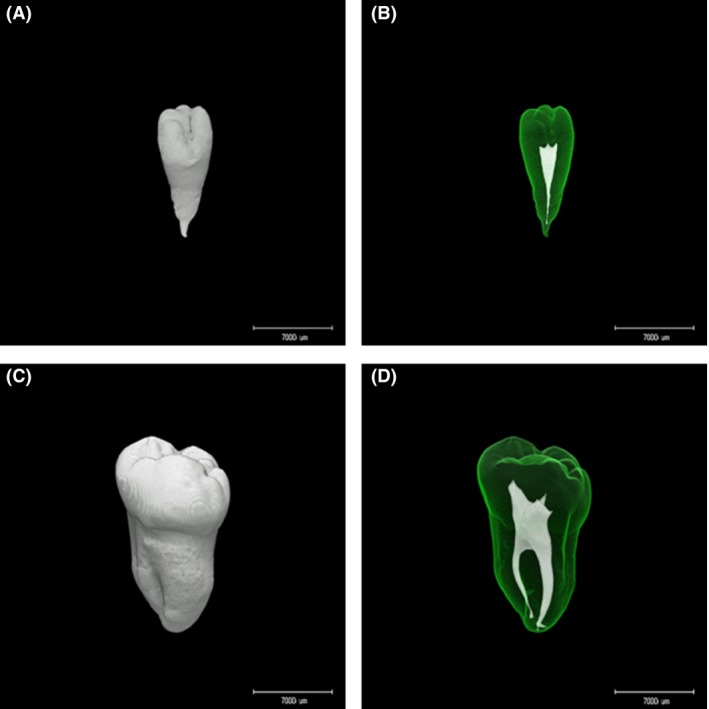
(A) Micro‐computerized tomographic image of microdontia of the maxillary third molar in a 32‐year‐old male patient. (B) Three‐dimensional representation of the root morphology of this microdontia. (C) Micro‐computerized tomographic image of a normal maxillary third molar in a 41‐year‐old male individual. (D) Three‐dimensional representation of the root morphology of this normal tooth. The tooth size in (A) is smaller than that in (C).

Answer: Microdontia.

Partially impacted maxillary third molars with pericoronitis were extracted from 32‐year‐old and 41‐year‐old male patients. Scanning was performed using an X‐ray micro‐CT system (SMX‐90; Shimadzu, Kyoto, Japan), and each specimen was imaged to reconstruct the structure 1.

Microdontia is a condition in which teeth are abnormally small. The incidence of microdontia reported is 1–8% condition 2. Figure 1A shows a micro‐computerized tomographic image representing microdontia of the whole tooth. Figure 1B and D are three‐dimensional representations of the root canal morphology of a tooth affected by microdontia and a normal tooth, respectively. These images show that microdontia appears normal in every respect, except for the tooth size. However, microdontia anomalies have been associated with malocclusion. Maxillary lateral incisors were the most affected by microdontia, and the next tooth that can be affected is the third molars. The third molars were the teeth most affected by impaction. Anomalies can cause esthetic and functional damages. Thus, computed tomography images are useful guides for the early detection and successful treatment of anomalies such as microdontia.

## Conflict of Interest

None declared.

## Authorship

TI: designed the project and wrote the draft of this manuscript. MS: collected and analyzed data and created the figures. FN and TM: aided in manuscript writing and editing. All authors have read and approved the final manuscript.
